# A latent profile analysis of category characteristics of learning engagement, achievement motivation and its influencing factors among Chinese medical students

**DOI:** 10.3389/fpsyg.2025.1619935

**Published:** 2025-09-19

**Authors:** Xiaoguang Wu, Wenxiang Shi, Xuemeng Song, Siyu Di

**Affiliations:** ^1^Clinical College of Anhui Medical University, Hefei, Anhui, China; ^2^Health Humanities Research Innovation Team, Clinical College of Anhui Medical University, Hefei, Anhui, China

**Keywords:** learning engagement, achievement motivation, career calling, latent profile analysis, medical students

## Abstract

**Purpose:**

This study aimed to investigate the heterogeneity of learning engagement and achievement motivation among medical students, understand the role of career calling in predicting the subcategories of learning engagement and achievement motivation among medical students, and provide empirical evidence for medical education reform.

**Methods:**

Latent profile analysis was used to investigate the latent characteristic patterns of learning engagement and achievement motivation, while polynomial logistic regression was used to investigate the predictive role of a sense of career using the latent categories of learning engagement and achievement motivation. The Learning Engagement Scale, Achievement Motivation Scale, and Career Calling Scale were used to survey students in their freshman and junior years (*n* = 1930) at a medical undergraduate college in Anhui Province, China.

**Results:**

(1) Positive associations were found between achievement motivation, learning engagement, and sense of career calling among medical students. (2) Five latent profiles of medical students’ learning engagement and achievement motivation were identified: “avoidant learners,” “negative learners,” “positive learners,” “enjoyable learners,” and “excessive learners.” The vast majority of medical students were classified as “avoidant learners” or “negative learners.” This suggests that the current level of learning engagement among medical students is low. (3) Medical students’ perceptions of their career calling have a considerable impact on the latent categories of learning engagement and achievement motivation. Specifically, “positive learners” have the strongest sense of career calling, followed by “excessive learners,” “enjoyable learners,” and “avoidant learners,” and the lowest by “negative learners.”

**Conclusion:**

Heterogeneity appears in medical students’ learning engagement and achievement motivation, with clear categorical traits and a strong correlation with career calling. The level of medical students’ learning engagement can be improved by stimulating their achievement motivation and increasing their career calling.

## 1 Introduction

Learning engagement (LE) is defined as a learner’s prolonged, positive emotional and mental state of fulfillment during the learning process, characterized by vigor, devotion, and concentration (Schaufeli et al., 2002). Learning engagement influences not only an individual’s willingness to study but also the level of academic accomplishment and ability that an individual can obtain (Kuh, 2009). For medical students, the level of learning engagement not only determines the systematicity of their theoretical knowledge and proficiency of their practical skills but also ensures medical safety and the credibility of the profession. The importance of learning engagement of medical students runs through the whole career cycle from “student” to “doctor” and ultimately affects the life and health safety of patients (Li Y. et al., 2024). Therefore, the level of learning engagement of medical students is directly related to the quality of future medical education and public health services. However, countries such as China (Huang et al., 2023), Chile (Gómez et al., 2015), Portugal (Abreu Alves et al., 2022), Canada (Babenko et al., 2018), the Netherlands (Wissing et al., 2022), and Spain (Urrizola et al., 2023) assessed the level of learning engagement of medical students based on mean scores using the center-of-variable method. The results showed that the learning engagement of medical students in each country was at a medium level, and there was considerable room for improvement. Moreover, existing studies lack further exploration of the development model and effective enhancement path of medical students’ learning engagement. In summary, the present study adopted an individual-centered approach, which was used to reveal the different developmental patterns that exist in the learning engagement of medical students that may have been overlooked by the variable-centered approach. This study also aimed to understand the role of career calling in predicting the developmental patterns of medical students and to provide a factual basis for improving the learning engagement of medical students and for carrying out reforms in medical education.

According to Peng (2018), motivation is the psychological inclination or inner drive that propels and maintains an organism’s behavior and guides it toward a specific objective. According to Atkinson (1957), there are two types of motivation: the motivation to approach success (MS), which aims to maximize personal satisfaction and involves positive evaluation outcomes of expectations, and the motivation to avoid failure (MF), which minimizes personal suffering and involves expectations of negative evaluation outcomes. Consequently, it is maintained that an individual’s subjective anticipation of success or failure, known as achievement motive (AM), is a consistent personal trait. Additionally, learning engagement encompasses the behavioral, emotional, cognitive, and other traits that people exhibit during the learning process, all of which are strongly linked to their drive to achieve their goals. Learning engagement and achievement motivation are positively correlated (Luo et al., 2023; Sun et al., 2023; Wang and Dong, 2022). When examining the relationship between learning engagement and achievement motivation in Chinese adolescents, Li X. et al. (2024a) discovered a strong positive correlation between the two. In particular, students’ level of learning engagement rose in proportion to their level of achievement motivation. Similarly, Luo et al. (2023) found the same result when investigating the relationship between learning engagement and achievement motivation among Chinese college students. To satiate the internal need for academic success and prevent academic failure, students will be motivated to exhibit higher levels of learning engagement throughout the learning process by achievement motivation, an internal driving force that pushes people to pursue success or avoid failure (Wang and Dong, 2022).

According to self-worth theory, people have an inherent urge to protect their sense of value against danger (Covington, 1984). Different actions are taken to safeguard self-worth when it is threatened by the outside world. Students share this urge to safeguard their self-worth throughout the learning process, and they do so by putting in greater effort, erecting obstacles for themselves, and using other tactics to prevent failures. In addition, Covington, in conjunction with the theory of achievement motivation (Atkinson, 1957), suggests that there are four distinct types of students with various forms of achievement motivation: “high tendency and high avoidance,” “high tendency and low avoidance,” “low tendency and high avoidance,” and “low tendency and low avoidance.” To further understand the variations in students’ motivation and performance, “tendency” refers to the motivation to succeed, while “avoidance” refers to the motivation to avoid failure. Synthesizing existing theoretical and empirical studies, there is a close connection between achievement motivation and learning engagement. Therefore, when exploring the developmental pattern of the learning engagement of medical students, this study should include their achievement motivation in the scope of examination as well as explore the developmental pattern of the learning engagement and achievement motivation of medical students.

According to (Dik et al., 2009), career calling (CC) is the capacity of an individual to pursue a job in a way that results in the realization or acquisition of a feeling of meaning in life and the association of occupational actions with positive affective experiences and values. Individuals with a sense of career calling go beyond using their careers as a way to gain income, and further link their careers to the realization of their life’s value (Hall and Chandler, 2005). According to self-determination theory, an individual’s conduct is determined by their own self-determination, meaning that when they are completely aware of their own requirements and external information, they can adopt any type of behavior (Gagné and Deci, 2005). High-caliber medical students should possess not only exceptional professional skills but also a strong sense of identification with their field and professional ethics (Zhao et al., 2024). When medical students have a strong sense of career calling, they hold a strong sense of purpose and learn the knowledge and skills needed by medical workers in a meaningful way. This is when learning behaviors are in line with their self-determined tendencies, and their level of learning engagement will naturally increase. (Wang et al., 2023). In addition, career calling is a strong feeling and motivation experienced by individuals in engaging in career behaviors, which encourages individuals to engage in their careers with positive attitudes. Achievement motivation is the internal motivation of individuals to continuously pursue higher standards, which motivates them to make continuous efforts to meet the needs of approaching success and avoid failure. Therefore, individuals with a high sense of career calling have a stronger achievement motivation (Yang et al., 2022). However, most existing studies have focused on variables to examine the relationship between variables such as learning engagement, achievement motivation, and career calling, and have not addressed the existence of differential effects between different developmental patterns of learning engagement, achievement motivation, and career calling. Therefore, this study further investigates whether career calling can be used as an antecedent variable to predict the categories to which the learning engagement and achievement motivation of medical students belong.

An individual-centered study technique called latent profile analysis (LPA) further divides people into subgroups according to the variations in their responses to extraneous variables (Wang J. et al., 2024). The objectivity of latent profile analysis in evaluating categorical indicators eliminates, to the greatest extent possible, the high intra-category heterogeneity caused by subjective categorization. It also captures group heterogeneity that is not visible in variable-centered studies, in contrast to previous studies that primarily used variable-centered perspectives with individual mean scores to understand the performance of individuals on various dimensions and then analyze the relationship between variables (Chen et al., 2022; Huang and Zhang, 2024). Furthermore, by using latent profile analysis (LPA) to profile medical students’ learning engagement and achievement motivation, examining the interactions between various aspects of these factors to create latent category differences, and using this as the foundation for classifying medical students’ learning engagement and achievement motivation, the relationships between various aspects can be further investigated (Li et al., 2021). Thus, this study sought to use LPA to investigate the latent categories and developmental features of medical students’ learning engagement and achievement motivation, examine the demographic distribution of various learning engagement and achievement motivation categories, comprehend the influence of career calling on various medical student categories, and offer empirical support for medical education reform.

Synthesizing the results of existing theoretical and empirical studies, this study proposes the following hypothesis:

1. Career calling can significantly and positively predict the levels of achievement motivation and dimensions of learning engagement among medical students.
2. Heterogeneity exists among the dimensions of learning engagement and achievement motivation of medical students, and there are significant differences in the dimensions of learning engagement and achievement motivation among different subcategories of medical students.
3. Career calling significantly predicted the latent categories of learning engagement and achievement motivation of medical students; that is, the higher the career calling, the less likely medical students were to be categorized into subcategories with lower learning engagement and achievement motivation.

## 2 Materials and methods

### 2.1 Participants

A convenience sampling method was used to select a medical university in Anhui Province, China, to distribute the e-questionnaires. An electronic questionnaire was developed using an online platform to collect data. The content included demographic information, the Learning Engagement Scale for College Students, the Achievement Motivation Scale, and the Career Calling Scale. To protect the privacy of the participants, the e-questionnaire did not contain any content related to personal information. To minimize the effect of social expectation error, the e-questionnaire began with a reminder that the participation of the study was anonymous, that the participants were required to answer according to their own reality, and that the findings would be used only for scientific research. Electronic questionnaires were then distributed to each class by professionally trained personnel who prompted students to read the instructions to ensure informed consent. To avoid missing data from the questionnaire, all questions were set as mandatory, and the electronic questionnaire could only be submitted after all the questions were completed.

A total of 2,100 questionnaires were returned. Excluding invalid questionnaires with regular answers and omissions, 1930 valid questionnaires were included in the analysis, with a validity rate of 91.9%. Among them, 756 (39.0%) were males and 1,174 (61.0%) were females. There were 608 (31.5%) students in the first grade, 1,034 (53.6%) in the second grade, and 288 (14.9%) in the third grade. The mean age was 20.11 (SD = 1.66) years old. There were 1,084 (56.2%) urban students and 846 (43.8%) rural students. There were 525 (27.2%) only children and 1,405 (72.8%) non-only children. The educational level of fathers and mothers was 15.2 and 26.5% for those in elementary school or below, 39.1 and 36.0% for those in junior high school, 31.2 and 28.3% for those in high school or middle school, and 14.5 and 9.1% for those in college or bachelor’s degree or above, respectively.

The study was approved by the local ethics committee (approval number: LCYXY00003), and all students provided informed consent. The recruitment period for this study was from September 20, 2023, to November 20, 2023.

### 2.2 Materials

#### 2.2.1 Learning engagement

The Learning Engagement Scale for College Students developed by Liao (2011) was used in this study. The scale is divided into three dimensions: behavioral, cognitive, and emotional engagement. Among them, behavioral engagement (BE) refers to the specific performance of college students in class, out of class, and during practice. Cognitive engagement (CE) refers to the learning methods adopted by college students based on their self-knowledge in the learning process. Emotional engagement (EE) refers to the subjective emotional experience of college students in the learning process. The scale includes 20 items. Participants answered each item on a five-point Likert scale (1 = totally incompatible, 5 = totally compatible). The higher the total score on the scale, the higher the level of learning engagement. In this study, the Cronbach’s *α* of the scale was 0.93, and the Cronbach’s α of the subscales were BE = 0.86, CE = 0.93, and EE = 0.88. The structural validity of this study was acceptable (χ^2^/*df* = 3.23, TLI = 0.97, CFI = 0.98, RMSEA = 0.06, SRMR = 0.03). (Example question: I often read materials related to my profession).

#### 2.2.2 Achievement motivation

The Achievement Motivation Scale, revised by Ye and Hagtvet (1992), was used in this study. The scale comprises 30 questions. The scale is divided into two dimensions: Motivation to Approach Success (MS) and Motivation to Avoid Failure (MF), with 15 questions each. In particular, motivation to approach success is associated with achieving success and involves positive outcome expectations. Motivation to avoid failure is linked to preventing failure and involves negative outcome expectations. Participants answered each item on a four-point Likert scale (1 = totally incompatible, 4 = totally compatible). Higher scores indicate that students are more motivated to approach success and avoid failure than lower scores. The total score for achievement motivation is the score for motivation to approach success minus the score for motivation to avoid failure. The Cronbach’s *α* of the scale in this study was 0.88, and the Cronbach’s α of the subscales were MS = 0.92 and MF = 0.92, respectively. The structural validity was acceptable (χ^2^/*df* = 7.63, TLI = 0.89, CFI = 0.91, RMSEA = 0.07, SRMR = 0.05). (Example: I get very excited and happy when I am faced with a problem that I am not sure I can overcome).

#### 2.2.3 Career calling

The Career Calling Scale in Chinese culture developed by Zhang et al. (2015) was adopted. The scale consists of 11 questions. The scale is divided into three dimensions: altruism, guiding force, and meaning and purpose. Altruism emphasizes the desire to help, serve others or the community, and give of oneself. Guiding force emphasizes that career calling has a guiding force or major task, so that people must accept it and try to fulfill it. Meaning and purpose emphasize the integration of an individual’s occupational roles with meaning, life purpose, and interest in life. Participants answered each item on a five-point Likert scale (1 = completely disagree, 5 = completely agree). Except for question 3, which was scored in reverse order. Higher scores on each question indicate a higher career calling. In this study, Cronbach’s *α* for the scale was 0.86. The Cronbach’s α for the three dimensions were 0.74 (Altruism), 0.89 (Guiding force), and 0.89 (Meaning and purpose). The structural validity was acceptable (χ^2^/*df* = 6.35, TLI = 0.98, CFI = 0.99, RMSEA = 0.05 SRMR = 0.03). (Example question: I want to pursue a career that will benefit others.)

### 2.3 Data processing

The study used SPSS 21.0 and M-plus 8.1 software to process the data. First, the data were analyzed for reliability using SPSS to test the scale reliability. Second, common method bias tests, descriptive statistical analyses, and standardization were performed. Next, a latent profile analysis of achievement motivation and learning engagement was conducted using M-plus.

The standardized scores of learning engagement in the three dimensions of behavioral, cognitive, and emotional engagement, and the standardized scores of achievement motivation in the two dimensions of motivation to approach success and failure-avoidance motivation were used as observational variables to establish a latent profile model of learning engagement and achievement motivation of medical students. The number of latent categories was gradually increased from one to six, after which the six model fits were compared and the optimal model was selected.

Latent profile analysis requires a combination of AIC, BIC, aBIC, Entropy, LMRT, and BLRT to determine the number of latent categories to determine the classification effect. Lower values of AIC, BIC, and aBIC indicate a better model fit (Morgan, 2015). Entropy values range from 0 to 1, with values greater than 0.80 when the model classification accuracy is greater than 90%, and the average probability takes a value between 0 and 1, with values closer to 1 indicating a more plausible classification (Tein et al., 2013). The LMRT and BLRT corresponded to the *p*-value reaching a significant level (*p* < 0.05), indicating that the K model was superior to the K-1 model (Masyn, 2013). It is worth noting that the choice of the number of categories should take into account the proportion of subjects in each category, which should be at least 1% of the total sample size (Jung and Wickrama, 2008). Finally, career calling was analyzed as an antecedent variable using multinomial logistic regression.

## 3 Results

### 3.1 Common method bias test

A common method bias test was conducted using the Herman one-way test, and an unrotated exploratory factor analysis was performed on 61 items. The results showed that eight factors had eigenvalues greater than 1, explaining 61.47% of the variance. The highest-ranked factor explained 27.64% of the variance, which was below the 40% threshold. Therefore, the results of this study were less affected by common method bias.

### 3.2 Descriptive statistics and correlation analysis

The means and standard deviations of learning engagement, achievement motivation, and their dimensions are shown in Table 1. Pearson correlation analysis showed a significant correlation between the dimensions of achievement motivation, learning engagement, and career calling.

**Table 1:** Descriptive statistics and correlation analysis results of learning engagement and achievement motivation dimensions with career calling (*n* = 1930).

Variables	*M*	*SD*	1	2	3	4	5
1. MF	3.34	0.64	—				
2. MS	3.41	0.63	−0.06^*^	—			
3. BE	3.14	0.71	−0.09^**^	0.51^**^	—		
4. CE	3.27	0.69	−0.11^**^	0.51^**^	0.80^**^	—	
5. EE	3.28	0.69	−0.11^**^	0.51^**^	0.72^**^	0.80^**^	—
6. CC	3.77	0.54	0.06^*^	0.59^**^	0.46^**^	0.43^**^	0.43^**^

*p < 0.05, **p < 0.01, ***p < 0.001. MS: motivation to approach success; MF: motivation to avoid failure; BE: behavioral engagement; CE: cognitive engagement; EE: emotional engagement; CC: career calling.

### 3.3 Latent profile model of learning engagement and achievement motivation of medical students

The results showed that Log, AIC, BIC, and aBIC showed a gradual decreasing trend as the number of latent categories increased, implying that the fit of the latent profile model was improving. The entropy value was highest at six categories (0.911), followed by five (0.905) and four (0.896) categories, implying that six categories were better than five, and five were better than four. LMRT was not significant in the six categories, but was significant in five and four categories (*p* < 0.001). In terms of category probabilities, all models had category probabilities greater than 1%. In summary, the LMRT for the 6 categories model was greater than 0.05, indicating that the 6 categories model was not better than the 5 categories model. Except for the 6 categories model, the 5 categories model has smaller Log, AIC, BIC, and aBIC values, larger Entropy values, and all category probabilities are greater than 1%, as shown in Table 2. Finally, the 5 categories model was selected as the optimal model by combining the accuracy and parsimony requirements for model selection.

**Table 2:** Fitting information for latent profile analysis of learning engagement and achievement motivation of different medical students.

MODEL	Log(L)	AIC	BIC	aBIC	Entropy	LMRT(*p*)	Category probability (%)
1	−13690.26	27400.51	27456.17	27424.40			
2	−12499.92	25031.83	25120.88	25070.05	0.83	0.00	69.3/30.7
3	−11901.56	23847.12	23969.56	23899.67	0.88	0.00	11.9/64.9/23.2
4	−11376.98	22809.95	22965.78	22876.82	0.90	0.00	9.9//30.0/6.7/53.4
**5**	**−11250.18**	**22568.35**	**22757.57**	**22649.55**	**0.91**	**0.00**	**53.7/9.4/2.1/5.1/29.7**
6	−11128.48	22336.95	22559.56	22432.48	0.91	0.36	1.0/5.2/29.3/51.2/11.3/2.0

Log(L): loglikelihood; AIC: Akaike Information Criterion; BIC: Bayesian Information Criterion; aBIC: adjusted Bayesian Information Criterion; LMRT: Lo–Mendell–Rubin Adjusted LRT Test. Bold values indicate that Model 5 is the optimal model.

The mean probabilities of participants being assigned to the five latent categories are shown in Table 3. For every medical student, the mean probability of being assigned to that group was above 0.90, and the mean probability of being assigned to the other groups was below 0.10, further validating the accuracy of the 5 categories model.

**Table 3:** Mean probability of attribution for each latent category of study subjects (rows).

Category	*N*	Percent	Attribution probability				
			Profile 1	Profile 2	Profile 3	Profile 4	Profile 5
Profile 1	1,037	53.7%	0.94	0.02	0.00	0.00	0.04
Profile 2	181	9.4%	0.05	0.95	0.00	0.00	0.00
Profile 3	40	2.1%	0.00	0.00	0.94	0.04	0.02
Profile 4	98	5.1%	0.00	0.00	0.04	0.91	0.05
Profile 5	574	29.7%	0.07	0.00	0.00	0.01	0.92

**Figure 1:**
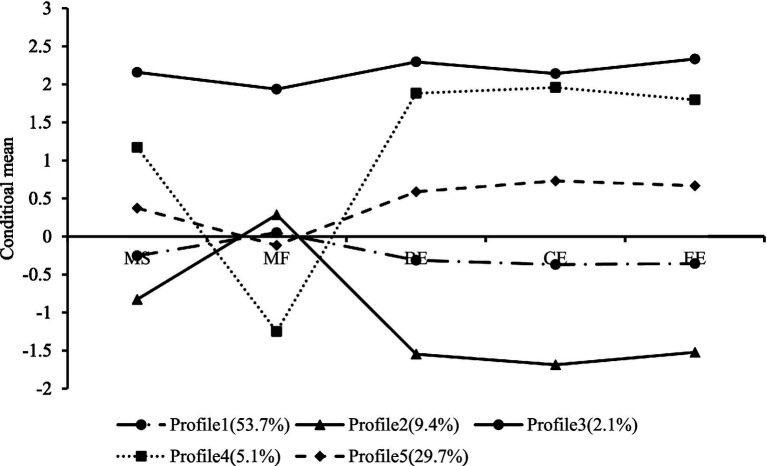
Mean scores of standardized scores of 5 latent categories of learning engagement and achievement motivation of medical students on each dimension. MS: motivation to approach success; MF: motivation to avoid failure; BE: behavioral engagement; CE: cognitive engagement; EE: emotional engagement; Profile 1: negative learners; Profile 2: avoidant learners; Profile 3: excessive learners; Profile 4: enjoyable learners; Profile 5: positive learners.

In addition, the distribution of standardized scores of learning engagement of medical students in the dimensions of cognitive, emotional, and behavioral engagement and the distribution of standardized scores of achievement motivation in the dimensions of motivation to approach success and motivation to avoid failure are shown in Figure 1. The five latent categories were named according to the score characteristics of each entry in each category.

The mean standardized scores of profile1 on the dimensions of motivation for success and learning engagement were negative and close to zero, while the scores on motivation to avoid failure were positive and close to zero. This indicates that medical students in this category have a low desire for success, are more worried about facing failure scenarios, and have relatively low learning engagement. Therefore, they were named “negative learners,” whose learning engagement did not stem from the pursuit of success but from the fear of failure scenarios, totaling 1,037 students (53.7%). Profile 2 had the lowest mean standardized scores for motivation for success and learning engagement, but higher scores for failure avoidance. This indicates that medical students in this category have the least motivation to pursue success, the highest internal motivation to avoid failure, and the lowest level of learning engagement. Therefore, they were named as “avoidant learners” with 181 students accounting for 9.4% of the total. Profile 3 had the highest mean standardized scores for all dimensions of achievement motivation and learning engagement. This indicates that this group of medical students is both eager to achieve success and afraid of facing failure, and they work extra hard during the learning process. Therefore, they were named as “excessive learners” with 40 students accounting for 2.1% of the total. Profile 4 had the second highest mean standardized scores for motivation for success and learning engagement, and the lowest score for motivation to avoid failure. This indicates that medical students in this category are eager to achieve success, are not afraid to face failure, and are willing to put more effort into the learning process. Therefore, it is named as “enjoyable learners,” with a total of 98 students, accounting for 5.1%. The mean standardized scores of profile 5 were positive in the dimensions of motivation for success and learning engagement but negative in the dimension of motivation to avoid failure. This indicates that students in this category have a higher desire for success, a lower fear of failure, and a higher level of learning engagement, which is the opposite of the “negative learners” score. Therefore, they were named as “positive learners,” with 574 students accounting for 29.7%. See Figure 1.

### 3.4 Differences between medical students with different types of learning engagement and achievement motivation on each dimension

To determine whether there is heterogeneity among medical students with different achievement motivation and learning engagement, the scores of the five latent categories of medical students on achievement motivation and learning engagement and their various dimensions were analyzed for differences. A multivariate ANOVA was conducted with the latent categories of achievement motivation and learning engagement as the independent variables, and motivation to approach success, motivation to avoid failure, cognitive engagement, behavioral engagement, and emotional engagement as the dependent variables. The results are shown in Table 4, which shows significant differences in the dimensions of achievement motivation and learning engagement among the five groups of medical students. Wilks’ *λ* = 0.10, *F*(20, 6,372) = 322.07, *p* < 0.001, *η*^2^ = 0.44, specifically in the motivation to approach success [*F*(4, 1925) = 215.56, *p* < 0.001, *η*^2^ = 0.31], motivation to avoid failure [*F*(4, 1925) = 100.32, *p* < 0.001, *η*^2^ = 0.17], behavioral engagement [*F*(4, 1925) = 1065.21, *p* < 0.001, *η*^2^ = 0.69], cognitive engagement [*F*(4, 1925) = 2105.87, *p* < 0.001, *η*^2^ = 0.81], and emotional engagement [*F*(4, 1925) = 1233.12, *p* < 0.001, *η*^2^ = 0.72] were significantly different. Furthermore, the least significant difference method was used to compare the differences between different types of achievement motivation and learning engagement of medical students on the dimensions. It was found that there were significant differences between the five types of medical students on the dimensions of motivation to approach success, motivation to avoid failure, cognitive engagement, behavioral engagement, and emotional engagement (*p* < 0.001). The results suggest that the latent categorization of learning engagement and achievement motivation of medical students in this study helps to differentiate achievement motivation and learning engagement among different categories of medical students, and that the categorization of the five categories has validity.

**Table 4:** Descriptive data and test of difference (*M ± SD*) for each dimension of achievement motivation and learning engagement for medical students in each latent category.

Variables	Latent profiles of achievement motivation and learning engagement among medical students					*F*	*η^2^*	LSD
	P1	P2	P3	P4	P5			
MS	−0.26 ± 0.79	−0.82 ± 1.00	2.21 ± 0.50	1.17 ± 1.07	0.37 ± 0.81	215.56^***^	0.31	P2 < P1 < P5 < P4 < P3
MF	0.05 ± 0.82	0.29 ± 1.00	2.02 ± 0.70	−1.21 ± 1.26	−0.12 ± 0.98	100.32^***^	0.17	P4 < P5 < P1 < P2 < P3
BE	−0.32 ± 0.52	−1.59 ± 0.64	2.30 ± 0.56	1.90 ± 0.68	0.59 ± 0.57	1065.21^***^	0.69	P2 < P1 < P5 < P4 < P3
CE	−0.38 ± 0.44	−1.72 ± 0.55	2.13 ± 0.29	1.97 ± 0.33	0.75 ± 0.40	2105.87^***^	0.81	P2 < P1 < P5 < P4 < P3
EE	−0.36 ± 0.50	−1.57 ± 0.70	2.33 ± 0.39	1.80 ± 0.64	0.68 ± 0.51	1233.12^***^	0.72	P2 < P1 < P5 < P4 < P3

*p < 0.05, **p < 0.01, ***p < 0.001.

### 3.5 The effect of career calling on different types of learning engagement and achievement motivation of medical students

Using career calling as the antecedent variable, polynomial logistic regression was used to determine whether career calling could influence the categories of achievement motivation and learning engagement to which medical students belong. The results showed that career calling had a significant effect (*p* < 0.001) on the classification of latent categories of learning engagement and achievement motivation of medical students, as shown in Table 5. This suggests that career calling can help predict the categories of achievement motivation and learning engagement to which medical students belong. Compared to Profile2, a higher sense of career calling reduces the incidence of individuals belonging to Profile1 by 97.4% [(e^0.68^–1) × 100%]. The higher the individual’s career calling, the more likely the individual was to be categorized as Profile 5, followed by Profile 3, Profile 4, and finally Profile 2, Profile 1.

**Table 5:** Results of polynomial logistic regression with learning engagement and latent categories of achievement motivation as dependent variables.

Profiles	Career calling			
	*β*	*SE*	*OR*	*p*
P1 vs. P2	−0.68	0.13	0.51	<0.001
P1 vs. P3	−2.20	0.23	0.11	<0.001
P1 vs. P4	−1.51	0.16	0.22	<0.001
P1 vs. P5	−3.98	0.52	0.02	<0.001
P2 vs. P3	−1.52	0.18	0.22	<0.001
P2 vs. P4	−0.82	0.09	0.44	<0.001
P2 vs. P5	−3.30	0.51	0.04	<0.001
P3 vs. P4	0.70	0.17	2.00	<0.001
P3 vs. P5	−1.78	0.51	0.17	<0.001
P4 vs. P5	−2.47	0.49	0.08	<0.001

## 4 Discussion

### 4.1 Development pattern and main features of learning engagement and achievement motivation of medical students

To understand the developmental patterns of learning engagement and achievement motivation of medical students, this study aims to promote the reform of medical education and improve the quality of medical student cultivation. Based on the perspective of “individual-centeredness,” this study uses the LPA method to investigate the heterogeneity of learning engagement and achievement motivation among medical students. The results show that there are five latent categories of achievement motivation and learning engagement among medical students, namely, “avoidant learners,” “negative learners,” “positive learners,” “enjoyable learners,” and “excessive learners.”

“Avoidant learners” (9.4%) had the lowest scores on motivation to approach success, behavioral engagement, cognitive engagement, and emotional engagement, but the second-highest scores on motivation to avoid failure. This is similar to “low-tendency, high-avoidance” type of students (Covington, 1984). This result suggests that avoiding failure is more important than achieving success for “avoidant learners,” and their level of learning engagement is also the lowest. According to self-worth theory (Wigfield and Eccles, 2000), to effectively avoid the threat to their self-worth posed by failure, students may minimize the challenge to their self-worth in the event of failure by setting up self-barrier strategies. The “avoidant learners” in this study used strategies to reduce learning engagement to minimize the negative impact of possible failure on their self-worth. “Negative learners (53.7%) had the second lowest scores in motivation to approach success, behavioral engagement, cognitive engagement, and emotional engagement, while motivation to avoid failure was positive. From Covington’s perspective, this is similar to the “low-tendency, middle-avoidance” type of student (Covington, 1984). This suggests that “negative learners” have higher motivation to pursue success and lower motivation to avoid failure than “avoidant learners.” As a result, they may be less influenced by self-barrier strategies, and therefore are able to maintain a low level of learning engagement.

“Positive learners” (29.7%) scored positively on motivation to approach success, behavioral engagement, cognitive engagement, and emotional engagement, and negatively on motivation to avoid failure, which was in the middle of the scale. From Covington’s perspective, this is similar to the “moderate-tendency, low-avoidance” type of student (Covington, 1984). The results show that contrary to “negative learners,” “positive learners” have more desire to approach success than fear of failure, which motivates them to maintain a moderate level of learning engagement. The “enjoyable learners” (5.1%) had the second-highest scores on motivation to approach success, behavioral engagement, cognitive engagement, and emotional engagement, and the lowest scores on motivation to avoid failure. This is consistent with the “high-tendency, low-avoidance” type of students (Covington, 1984). These students desire to succeed and are not afraid to fail, are interested in learning, and have an extremely high level of learning engagement. The “excessive learners” (2.1%) scored highest on motivation to approach success, motivation to avoid failure, behavioral engagement, cognitive engagement, and emotional engagement. This is similar to the “high-tendency, high-avoidance” type of students. This type of students is both eager to succeed and afraid of failure, love and hate for learning, in a contradictory state.

Among the five latent categories, “negative learners” and “avoidant learners” accounted for 63.1% of the total, and the level of learning engagement of medical students in these two categories was below the medium level. This is consistent with existing studies (Huang et al., 2023; Shida, 2024). There is still much room for improvement in the current learning engagement of medical students. This issue is closely related to the motivation of medical students (An et al., 2022; Cazan, 2015). It can be found that the motivation to approach success is lower than the motivation to avoid failure in both categories of “negative learners” and “avoidant learners,” and it can be seen that the achievement motivation of these two categories of medical students is negative. In addition, the lowest learning engagement of “avoidant learners” has a much lower motivation to approach success than motivation to avoid failure; therefore, it can be seen that the achievement motivation level of this type of medical student is the lowest.

To summarize, we can try to improve the achievement motivation of “negative learners” and “avoidant learners” to solve the problem of insufficient learning engagement. On the one hand, we can reduce the fear of academic failure among medical students and lower their motivation to avoid it. On the other hand, we can increase medical students’ desire for academic success and enhance their motivation to approach success. In this way, we can promote the gradual transformation of “negative learners” and “avoidant learners” to “active learners” and “enjoyable learners,” and enhance their learning motivation. As for “excessive learners,” we need to reduce their fear of academic failure and the impact of academic anxiety to facilitate their transformation into “enjoyable learners.”

### 4.2 Differences in the dimensions of latent profile of learning engagement and achievement motivation among different medical students

This study found significant differences in the dimensions of achievement motivation and learning engagement among the five groups of medical students. This result indicates that the five categories of latent profiles can effectively discriminate the different developmental patterns of learning engagement and achievement motivation of medical students and understand the heterogeneous characteristics that exist in the learning engagement and achievement motivation of medical students. This is in line with previous studies. Chen et al. (2024), when investigating the learning engagement of 70,678 Chinese college students, similarly found that the learning engagement of college students can be categorized into five categories, which proves the reliability of the results of this study.

Compared with existing studies (Cano et al., 2024; Wang Q. et al., 2024), the present study included both achievement motivation and learning engagement of medical students, which can further understand the relationship between achievement motivation and learning engagement of medical students, and clarify the internal reasons for the different performance of medical students’ learning engagement. This study found that the latent category of learning engagement is closely related to the latent category of achievement motivation, and the greater the achievement motivation, the higher the level of learning engagement of medical students in the other four categories except for “excessive learners.”

Although there is a lack of direct research on the characteristics of the achievement motivation categories of medical students, the results of this study show that the achievement motivation and learning engagement characteristics of “excessive learners,” “enjoyable learners,” and “avoidant learners” are similar to those of “high-tendency, high-avoidance,” “high- tendency, low-avoidance,” and “low-tendency, high-avoidance” as proposed by Covington (1984). This further validates the reliability of the results of this study. In addition, this study did not find any latent category of medical students that matched the characteristics of “low-tendency, low- avoidance,” which may be due to the fact that the subjects of this study were all medical students in higher education. The subjects of this study had successfully passed the entrance examination for higher education and achieved a certain level of academic success. The “low-tendency, low-avoidance” students have no desire for academic success nor fear of academic failure, and their indifference to academics will ultimately lead to their inability to pass the university entrance examination and receive higher education, which naturally prevents them from being included in the study.

### 4.3 The effect of career calling on the latent categories of learning engagement and achievement motivation of medical students

The results of the study found that career calling had a significant effect on the latent categories of learning engagement and achievement motivation among medical students. The higher an individual’s career calling is, the more likely they will be categorized as “active learners” and “excessive learners,” followed by “enjoyable learners” and “avoidant learners,” and the least likely to be categorized as “negative learners.” The results showed that medical students with a high level of career calling also had a higher level of learning engagement and achievement motivation, and that career calling is important for medical students to improve their learning engagement and achievement motivation. This result is consistent with existing research showing a positive correlation between career calling and learning engagement (Huang and Zhang, 2024) and achievement motivation (Yang et al., 2022). However, it is worth noting that the effect of career calling on learning engagement and achievement motivation latent categories of medical students is not a simple linear relationship. The higher the career calling of medical students, the more likely they were to be categorized as “active learners,” who were lower in learning engagement than “excessive learners” and “enjoyable learners” and lower in achievement motivation than “enjoyable learners.”

The reasons for this performance may be related to the differences in motivation. First, because “enjoyable learners” are full of interest in learning, eager to achieve academic success, and do not fear academic failure, they are able to engage in learning spontaneously. Second, because the “excessive learners” are often in a state of ambivalence and tension, when facing learning tasks, they are forced to increase their learning engagement in order to avoid academic failure (Covington, 1984). Therefore, for both “enjoyable learners” and “excessive learners,” the type of achievement motivation allows them to maintain a high level of learning engagement without the need for a higher career calling (Chen et al., 2022). In contrast, “avoidant learners” have the lowest achievement motivation and are self-protective. After they feel a sense of career calling, this strong emotional experience may bring more pressure, and they may worry that they will not be able to achieve their goals and their self-worth will be jeopardized, which will strengthen their self-barrier strategies and reduce their learning engagement level (Yan and Lan, 2007). Finally, “negative learners” have the lowest career calling and “positive learners” have the highest career calling, and the two groups have opposite performance in achievement motivation and learning engagement. This implies that career calling helps “active learners” to maintain higher learning engagement and achievement motivation, and it can be used as an effective way to enhance the learning engagement and achievement motivation of “negative learners.”

## 5 Limitations and perspectives

Inevitably, this study had some limitations. First, the object of this study only includes medical students in the higher education stage, who have been screened by the higher education entrance examination, and their learning ability has been verified to a certain extent. Medical education should also include the vocational education stage, and the characteristics of learning engagement and achievement motivation of medical students in the vocational education stage should also be included in the scope of future studies to investigate whether there are differences in the characteristics of learning engagement and achievement motivation of medical students with different academic qualifications.

Second, this study used a convenience sampling method to select only a sample of medical students from a university in Anhui Province, China, which may have led to an under-representative sample and affected the external validity of the study results. In the future, the sampling scope can be further expanded by using multilevel sampling to cover medical student groups in multiple countries to enhance the external validity of the study’s results.

Finally, this study demonstrated that career calling was predictive of the categories of achievement motivation and learning engagement among medical students using a cross-sectional design. However, it is not clear how career calling affects the achievement motivation and learning engagement of medical students, and whether enhancing the career calling of “negative learners” can effectively change their performance in achievement motivation and learning engagement toward “positive learners” is a question that needs to be answered in the future. Whether enhancing the sense of professional mission of “negative learners” can effectively change their performance in achievement motivation and learning engagement toward “positive learners” needs to be verified through intervention studies in the future.

## 6 Conclusion

This study explored the relationship between career calling and the subcategories of learning engagement and achievement motivation of medical students based on latent profile analysis and reached the following conclusions:

1. The results of this study showed a positive correlation between motivation to approach success, failure-avoidance motivation, behavioral engagement, emotional engagement, and cognitive engagement, all of which were positively related to career calling. This indicates that career calling can significantly and positively predict the level of achievement motivation and learning engagement dimensions of medical students, which validates hypothesis 1.
2. The results of the study showed that there are five latent profiles of learning engagement and achievement motivation among medical students: “avoidant learners,” “negative learners,” “positive learners,” “enjoyable learners,” and “excessive learners.” Among them, “avoidant learners” and “negative learners” accounted for the majority of medical students. In addition, there were significant differences in the dimensions of learning engagement and achievement motivation among the five medical student subcategories. This indicates that there is heterogeneity in the dimensions of learning engagement and achievement motivation among medical students, with obvious categorization characteristics among subgroups, and that the level of learning engagement of the majority of medical students needs to be improved, which confirms Hypothesis 2. This suggests that attempts can be made to improve the level of learning engagement by increasing medical students’ desire to succeed and decreasing their fear of failure.
3. The results of this study showed that career calling had a significant effect on the classification of latent categories of learning engagement and achievement motivation among medical students. In particular, “active learners” had the highest career calling, followed by “excessive learners,” “enjoyable learners,” “avoidant learners,” and the lowest was “negative learners.” This indicates that career calling can effectively predict the latent categories of learning engagement and achievement motivation of medical students, and validates Hypothesis 3. This suggests that the level of learning engagement of “negative learners” can be improved by increasing their career calling so that they can be converted to “positive learners.”

## Data Availability

The raw data supporting the conclusions of this article will be made available by the authors, without undue reservation.
